# A Spatio-temporal Model of African Animal Trypanosomosis Risk

**DOI:** 10.1371/journal.pntd.0003921

**Published:** 2015-07-08

**Authors:** Ahmadou H. Dicko, Lassane Percoma, Adama Sow, Yahaya Adam, Charles Mahama, Issa Sidibé, Guiguigbaza-Kossigan Dayo, Sophie Thévenon, William Fonta, Safietou Sanfo, Aligui Djiteye, Ernest Salou, Vincent Djohan, Giuliano Cecchi, Jérémy Bouyer

**Affiliations:** 1 West African Science Service on Climate Change and Adapted Land Use, Climate Change Economics Research Program, Cheikh Anta Diop University, Dakar-Fann, Sénégal; 2 The Pan African Tsetse and Trypanosomiasis Eradication Campaign (PATTEC), Bobo-Dioulasso, Burkina Faso; 3 Ecole Inter Etats des Sciences et Médecine Vétérinaires de Dakar (EISMV), Dakar, Sénégal; 4 Veterinary Services Department of the Ministry of Food and Agriculture, Pong-Tamale, Ghana; 5 Centre International de Recherche-Développement sur l'Elevage en zone Subhumide (CIRDES), Bobo-Dioulasso, Burkina Faso; 6 CIRAD, UMR INTERTRYP, Montpellier, France; 7 West African Science Center on Climate Change and Adapted Land Use, Ouagadougou, Burkina Faso; 8 Direction Nationale des Services Vétérinaires, Pan African Tsetse and Trypanosomosis Eradication Campaign (PATTEC), Mali, Bamako, Mali; 9 Université Polytechnique de Bobo Dioulasso (UPB), Bobo Dioulasso, Burkina Faso; 10 Felix Houphouet Boigny University, National Institute of Public Health, Abidjan, Côte d'Ivoire; 11 Food and Agriculture Organization of the United Nations (FAO), Sub-regional Office for Eastern Africa, Addis Ababa, Ethiopia; 12 CIRAD, UMR CMAEE, Dakar-Hann, Sénégal; 13 CIRAD, UMR CMAEE, Montpellier, France; 14 INRA, UMR1309 CMAEE, Montpellier, France; 15 Institut Sénégalais de Recherches Agricoles (ISRA), Laboratoire National d'Elevage et de Recherches Vétérinaires (LNERV), LNERV, Dakar-Hann, Sénégal; IRD/CIRDES, BURKINA FASO

## Abstract

**Background:**

African animal trypanosomosis (AAT) is a major constraint to sustainable development of cattle farming in sub-Saharan Africa. The habitat of the tsetse fly vector is increasingly fragmented owing to demographic pressure and shifts in climate, which leads to heterogeneous risk of cyclical transmission both in space and time. In Burkina Faso and Ghana, the most important vectors are riverine species, namely *Glossina palpalis gambiensis* and *G*. *tachinoides*, which are more resilient to human-induced changes than the savannah and forest species. Although many authors studied the distribution of AAT risk both in space and time, spatio-temporal models allowing predictions of it are lacking.

**Methodology/Principal Findings:**

We used datasets generated by various projects, including two baseline surveys conducted in Burkina Faso and Ghana within PATTEC (Pan African Tsetse and Trypanosomosis Eradication Campaign) national initiatives. We computed the entomological inoculation rate (EIR) or tsetse challenge using a range of environmental data. The tsetse apparent density and their infection rate were separately estimated and subsequently combined to derive the EIR using a “one layer-one model” approach. The estimated EIR was then projected into suitable habitat. This risk index was finally validated against data on bovine trypanosomosis. It allowed a good prediction of the parasitological status (r^2^ = 67%), showed a positive correlation but less predictive power with serological status (r^2^ = 22%) aggregated at the village level but was not related to the illness status (r^2^ = 2%).

**Conclusions/Significance:**

The presented spatio-temporal model provides a fine-scale picture of the dynamics of AAT risk in sub-humid areas of West Africa. The estimated EIR was high in the proximity of rivers during the dry season and more widespread during the rainy season. The present analysis is a first step in a broader framework for an efficient risk management of climate-sensitive vector-borne diseases.

## Author Summary

African animal trypanosomosis (AAT) is a major constraint to sustainable development of cattle farming in sub-Saharan Africa. The habitat of the tsetse fly vector is increasingly fragmented owing to demographic pressure and shifts in climate, which leads to heterogeneous risk of transmission both in space and time. In Burkina Faso and Ghana, the most important vectors are riverine species that are more resilient to human-induced changes than savannah and forest tsetse species. Therefore, understanding the spatio-temporal distribution of AAT risk remains an important task in order to design effective disease management approaches. The model developed in this research provides a fine-scale picture of the dynamics of AAT risk in sub-humid areas of West Africa. The output of the model is a risk index, the entomological inoculation rate, and it was validated against bovine trypanosomosis data using regression analysis. Parasitological status of cattle was accurately predicted, serological status was positively correlated but less accurately, whereas clinical case was not related to EIR. Our results show that the risk was high in the proximity of rivers during the dry season and more widespread during the rainy season.

## Introduction

In sub-Saharan Africa, African animal trypanosomosis (AAT) is one of the main constraints to the sustainable development of cattle farming [1]. In recent years, the habitat of tsetse fly vector (genus *Glossina*) has undergone significant modifications due to demographic and climatic pressures. Landscape fragmentation is progressively reducing the geographic distribution and densities of tsetse, and is also affecting the epidemiology of the disease by reducing host, vector and parasite diversities [2]. In Burkina Faso and Ghana, climatic and human factors, such as cattle keeping and crop-farming, have altered the riverine landscapes over the last decades, leading to a fragmentation of gallery forests [3]. Two tsetse species remain in most of this region, namely *Glossina palpalis gambiensis* Vanderplank and *Glossina tachinoides* Westwood (Diptera: Glossinidae). Their presence and densities heavily depend on the ecotype of riverine vegetation and its degree of disturbance [4].

In Burkina Faso, several studies have investigated the impact of fragmentation on tsetse distribution and densities [4], as well as on population structure and dispersal [5,6]. A longitudinal survey investigated seasonal dynamics of tsetse and mechanical vectors of trypanosomoses in landscapes at various levels of fragmentation [6]. Environmental factors, namely temperature and relative humidity, appeared to structure tsetse distribution and densities quite differently to those of most species of mechanical vectors. Mean maximum temperature was also found to be highly correlated to the tsetse infectious rates [7]. Finally, the cyclical risk of AAT transmission was mapped during the dry and rainy seasons of the year 2005 using the entomological inoculation index, i.e. the product of tsetse apparent densities and their infection rate [8,9].

A spatio-temporal model of tsetse apparent densities was also developed in a few sites along the Mouhoun river, where a longitudinal monitoring of the parasitological status of cattle was conducted [10]. Finally, two recent national eradication initiatives with a regional dimension were undertaken in south-western Burkina-Faso and north-western Ghana under the umbrella of the Pan African Tsetse and Trypanosomosis Eradication Campaign (PATTEC), within which extensive baseline data on vector distributions and disease prevalence were generated [11,12].

By building on the above body of information, the present paper focuses on AAT risk assessment by developing a spatio-temporal statistical model of the entomological inoculation rate (EIR). EIR is a simplified index derived from vectorial capacity, which is directly correlated to the rate of transmission (R0) of a vectorial disease (see [13] for a detailed explanation). This index does not give the prevalence in cattle, but the risk for cattle that would enter a given area to become infected from a bite by cyclical vectors. Since we used cattle parasites only to calculate and model the infection rate in tsetse, the risk that we map here is specific to cattle. The use of a simplified index presents the benefit to avoid the multiplication of uncertainties for each parameter that finally reduces the predicting power of such an index [14]. A number of authors have demonstrated previously that EIR (or tsetse challenge) is well correlated to the incidence of trypanosomosis in animals (see [15–19]. This is the first time however that this risk index is mapped in space and time and linked to climatic variables. This index will help designing some future climate risk management mechanisms to control AAT. In particular, early warning system and potential index based insurance can be built using the output of this spatio-temporal modeling of AAT risk.

## Materials and Methods

### Study area

The study area in south-western Burkina Faso and north-western Ghana is located between latitude 9°23'- 15°5' N and longitude 0°29'- 5°31' W. The area is approximately 372,000 km^2^, and the main river is the Mouhoun/Black Volta. Mean monthly temperatures vary between a minimum of 18°C and a maximum of 36°C and annual precipitation between 250 and 1,170mm. The study area is constituted of Sudano-Guinean savannah in the south, Sudanian savannah in the central part and Sahelian savannah in the north [20].

### Entomological data

In Burkina Faso, tsetse eradication efforts, targeting the northern part of the Mouhoun river basin started in 2008 (http://www.pattec.bf). In Ghana, the eradication project started in 2010 [12]. During the feasibility studies of these projects, baseline entomological surveys were carried out and generated an important amount of data, such as tsetse apparent density and their trypanosome infection rates. Biconical traps were used in all surveys [21].

In Burkina Faso, for the PATTEC baseline survey, all traps were set for three days [12] and they were deployed following a grid-based approach, within grid cells of 10x10 km [22]. Within each grid cell, 13 traps were set in the most suitable sites, in particular along the rivers and riparian thickets.

We also used longitudinal data on tsetse densities and infection rates originating from a longitudinal survey conducted in Burkina Faso. In this survey, 13 traps were spaced by 100m and set along three sections of the Mouhoun river. Traps were kept in place for three days a month, for total duration of 18 months in 2006 and 2007 [23].

The last dataset from Burkina Faso are from a recent study in the southern party of the country where entomological surveys were conducted in Moussodougou and Folonzo [24]. In these surveys, 25 traps were deployed in each site for 5 days during the rainy and dry seasons 2011–2012.

In the PATTEC baseline entomological survey done in Ghana, traps were deployed every 200m for 24h along the main rivers in dry seasons of 2008 and 2009 [12].

In addition to this entomological data from Burkina Faso and Ghana, 25 biconical traps were set in Kalofo, in northern Côte d’Ivoire. This survey was conducted during the dry and rainy seasons 2012 [25]. The space between each trap was 200m along a transect and they were set for 5 days and collected daily [26].

Finally in Mali, we used data from a PATTEC baseline entomological survey conducted in 2000–2002 for the habitat suitability model only. In this study, traps were set every 1km for 24h along the rivers (Djiteye, personal communication, and data in S1 File).

S1 and S2 Figs present the entomological data used in the models that are also provided as supplementary materials (data in S1 and S2 Files).

### Parasitological and serological data

Data on bovine trypanosomosis originated from various sources and studies. In particular, the parasitological and serological statuses and the packed cell volume (PCV) of surveyed bovines were assembled. PCV is the proportion of red cells in the blood; it allows measuring the level of anemia in cattle. We used a threshold of 25% below which the animal was considered anemic [27]. Anemia is one of the main symptoms of AAT and it is considered to be correlated with most cattle productivity parameters [28]. In addition to data on trypanosomosis and anemia (PCV), information on sex, breed, and age of animals was also available. Three sources were used to generate the final dataset.

The first source is a cross-sectional survey carried out in the Boucle du Mouhoun region in Burkina Faso: 47 villages were selected and 2,650 cattle were sampled between September 2007 and November 2007. The study and experimental design have been previously described [11].

The second dataset is from a longitudinal survey conducted in southern Burkina Faso. Six villages were sampled and a total of 363 cattle were monitored every four weeks between June 2003 and June 2005 [23].

The last survey was performed between February 2008 and March 2008 in the Upper West Region of Ghana. In this cross-sectional study, the area was divided into 180 grid cells of 10x10 km and 36 cells were randomly selected. In each cell, 50 cattle were sampled giving a total of 1,800 cattle for the whole area [12].

For all of the above surveys, blood samples were obtained from each animal and the level of parasitaemia was scored using to the phase contrast buffy coat technique [29]. For the serological status, antibodies against *Trypanosoma vivax*, *T*. *congolense* and *T*. *brucei* were detected using the antibody enzyme-linked immunosorbent assay (ELISA) [30]. Finally, the PCV, a measure of anaemia, was recorded after centrifugation of blood samples. S3 Fig presents the location of the sampled herds and their serological prevalence. Data are provided in S3 File.

### Environmental data

**Fig 1:**
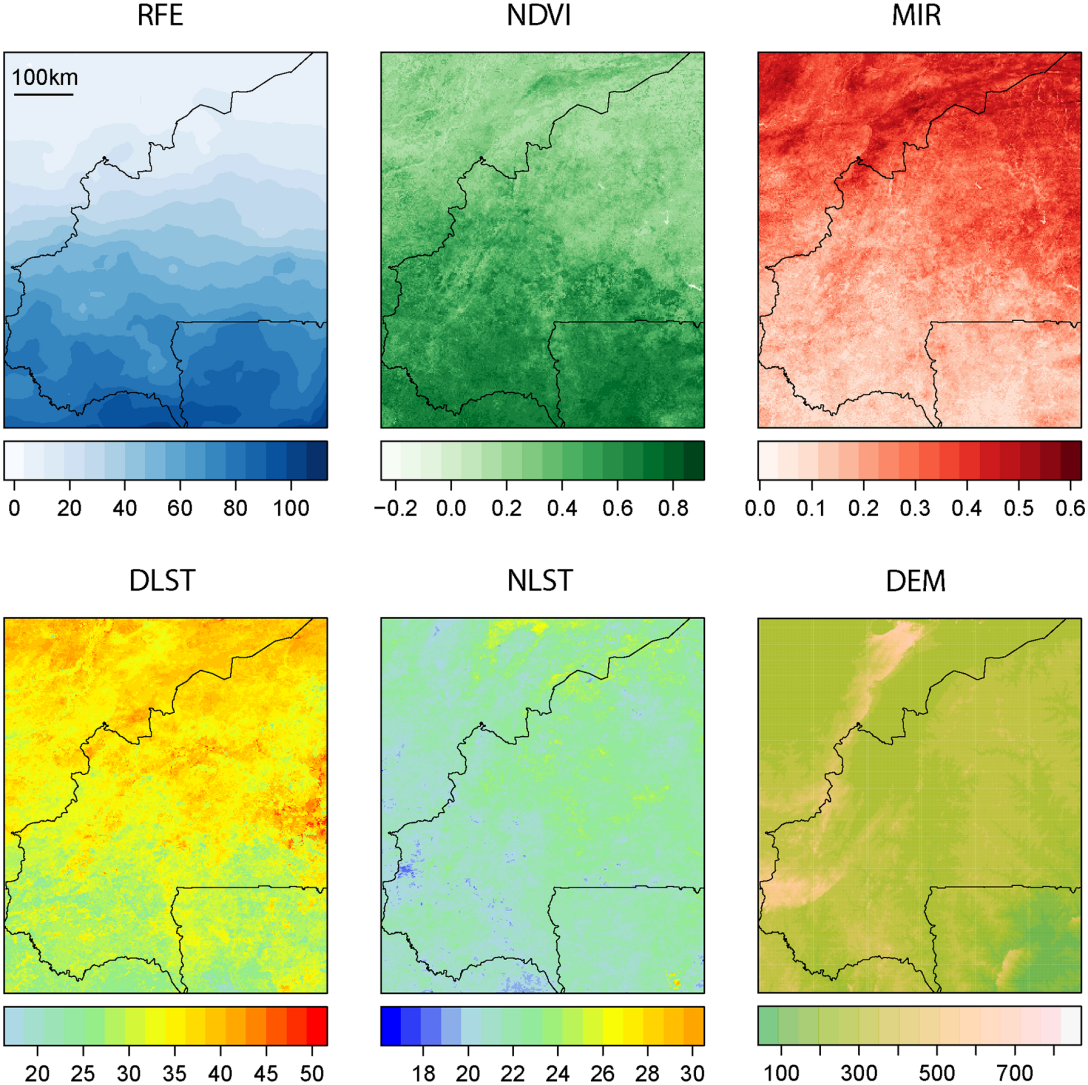
Remote sensing data from which environmental data was built. RFE (precipitation), NDVI (vegetation), MIR (vegetation), DLST (thermal), NLST (thermal) are time series of monthly raster grids from Jan. 2003 to Dec. 2013. DEM (topographic) is a static elevation model.

For the present study, a series of remote sensing data at high spatial and temporal resolution was used to assess the spatio-temporal risk of AAT (Fig 1 and Table 1). Firstly, Moderate-resolution Imaging Spectroradiometer (MODIS) data from the Terra and Aqua satellites were downloaded (http://e4ftl01.cr.usgs.gov/MOLT for Terra and http://e4ftl01.cr.usgs.gov/MOLA for Aqua). Daytime (DLST) and night-time land surface temperature (NLST) were extracted from MOD11A2/MYD11A2 temperature and emissivity MODIS products. DLST and NLST are used as proxies for both soil and air temperature, which play an important role in the epidemiology of AAT.

**Table 1:** Environmental data derived from remote sensing used for the analysis.

Variable name (extended)	Variable name (short)	Type	Source
Day Land Surface temperature	DLST	Thermal	MODIS
Night land surface temperature	NLST	Thermal	MODIS
Rainfall Estimate	RFE2	Precipitation	FAO
Normalized Differenced Vegetation Index	NDVI	Vegetation	MODIS
Middle Infra-Red	MIR	Vegetation	MODIS
Digital Elevation model	DEM	Topographic	SRTM
Cattle density	Cattle_density	Other	FAO

Both DLST and NLST data have a temporal resolution of eight days for each satellite (same composite daily data patched for both Terra and Aqua) and a spatial resolution of 1km. Low quality pixels were removed using the accompanying quality assessment layer and outliers were filtered using a variant of the boxplot algorithm [31]. The cleaned time series of DLST and NLST data was finally averaged monthly.

Monthly vegetation indices at 1km spatial resolution and monthly temporal resolution (MOD13A3/MYD13A3) were also downloaded and processed using a quality assessment layer. In particular, the Normalized Difference Vegetation Index (NDVI) and Middle Infrared (MIR) reflectance were selected to describe the vegetation condition in the study area.

Finally, a time series of dekadal gridded (11km spatial resolution) precipitation data product from FEWS-NET called Rainfall Estimator version 2 (RFE2) [32] were downloaded, downscaled (using bilinear downscaling) to match MODIS-based covariates spatial resolution (1km) and temporal resolution (monthly cumulated precipitation). We thus ended up with 11 years of monthly environmental variables (DLST, NLST, NDVI, MIR, and RFE) for the period up to—December 2013. However, the resulting dataset were still missing a few values due to cloud contamination, failure of some satellite instruments, and the data pre-processing scheme used (filtering of outliers and low quality pixels). Therefore, a spatio-temporal spectral analysis was used to fill the gaps. In particular, multivariate singular spectrum analysis was used because of its ability to capture the spatio-temporal dependence in the data and its excellent performance in comparison to other gap-filling routines when using similar spatio-temporal data [33,34]. In addition to these time series of remote sensing data, a digital elevation model (DEM) from the Shuttle Radar Topography Mission (SRTM) was used. The SRTM product at 1km spatial resolution was acquired through the CGIAR-CSI GeoPortal (http://srtm.csi.cgiar.org/). Lastly, a recently enhanced FAO cattle density layer was used [35]. This layer matches fairly recent statistics (2006 FAOstat data) and is characterized by a spatial resolution of 1km. This data was downloaded from the FAO Geonetwork website (http://www.fao.org/geonetwork/).

### Models

The main goal of the modelling exercise was to estimate the EIR in the study area using climatic and environmental data. EIR represents the number of infectious bites a host receives during a given period of time.

EIR, which is also known as tsetse challenge, is one of the most widely used and effective indicators of risk for tsetse-borne trypanosomosis [15].

The indicator is well known and widely used by malariologists to measure the intensity of malaria transmission [36]. Some efforts have been recently made to map EIR for malaria using similar spatio-temporal entomological data [37].

EIR is calculated as the product of tsetse apparent density and trypanosome infection rates of tsetse.

For a location *s* at a time *t*, we thus have:

$${\text{EIR}}_{\text{s},\text{t}}={\text{ADT}}_{\text{s},\text{t}}\times {\text{IR}}_{\text{s},\text{t}}$$

(ADT and IR are the apparent density per trap and infection rates respectively).

For this study, the statistical models were fitted separately for each one of the two layers constituting the EIR. This was necessary because the input data used originate from various sources, and, in particular, infection rates were not available for all samples. In order to maximize the use of all available data, we decided to compute separately the apparent density and infection rates, rather than fitting a single model for the observed EIR. For the rest of this analysis, the following components of the EIR were then considered:

- Tsetse habitat suitability.
- Tsetse apparent density per trap.
- Trypanosome infection rates in tsetse.

The first layer, habitat suitability, is not part of the mathematical definition of EIR, but it is always implied that we measure the risk of transmission where the vector occurs. Consequently, we first analyzed the habitat of the main tsetse vectors of AAT in the study area (i.e. *G*. *p*. *gambiensis* and *G*. *tachinoides*) before estimating and predicting EIR where the vectors can survive and transmit AAT.

### Tsetse habitat suitability

The first layer needed to map the risk index is the habitat suitability. We used this layer to determine the area where the vector of the disease can survive (the ecological niche). A statistical analysis of the habitat was carried out using correlative species distribution models. Occurrence data from already described entomological surveys were used as input. Characterization of the environment in the study area relied on the 11-year average, minimum, maximum, range and standard deviation of each spatio-temporal layer (DLST, NLST, NDVI, MIR, RFE), with the DEM added to the set of summarized variables.

The methodology used to predict tsetse habitat suitability is based on the framework developed in the Niayes areas (Senegal) using the Maximum Entropy model (MaxEnt) [38]. MaxEnt is one of the most widely-used species distribution models. It is a machine learning method based on the information theory concept of maximum entropy [39]. MaxEnt fits a species distribution by contrasting the environmental condition where the species is present to the global environment characterized by some generated pseudo-absence data, also called the background. The logistic output from MaxEnt is a suitability index that ranges between 0 (least suitable habitat) and 1 (most suitable habitat). It therefore gives us a quantitative indicator of the habitat preferences of the two tsetse species in the study area.

Moreover, to account for the sampling bias present in the entomological data, a gaussian kernel based grid that gives more weight to more densely sampled areas was constructed (S4 Fig).

In order to build this grid, a smoothing parameter is needed. Five parameters corresponding to the range of maximal dispersal distance of tsetse fly were used (2, 4, 6, 8, 10km) [40] to build five bias grids for the MaxEnt models [41,42]. Model complexity in the MaxEnt framework can be controlled using the beta regularization parameter. Five parameters (1, 1.5, 2, 3, 4) were used to fit a model for each parameter. Finally, we ended up with five regularization parameters and five bias grid (one for each smoothing parameter), resulting in twenty five models. Multi-model inference was then made using model averaging weighted by the AICc [43,44].

A model was fitted for each species and we created binary maps by setting the thresholds for presence that maximize the True Skill Score (TSS = sensitivity + specificity). These thresholds were 0.33 and 0.30 for *G*. *p*. *gambiensis* and *G*. *tachinoides* respectively. The final layer of tsetse habitat suitability for both species was obtained by combining the two previous layers: a pixel was considered as tsetse infested when it was infested by at least one species.

### Tsetse apparent density

The second layer of the risk index is the dynamic of the apparent density of tsetse flies, as measured using biconical traps, considered here as substitution hosts. The number of tsetse caught per trap per day is thus considered to be correlated to the relative density of tsetse to hosts. We predicted tsetse apparent density per trap (ADT) at a monthly temporal resolution and a spatial resolution of 1km2 using spatio-temporal statistical model fitted against the monthly temperature (DLST), vegetation (NDVI) and the DEM. A negative binomial model with spatial random effects was used. Negative binomial models can be seen as an extension of the classical Poisson regression to account for over-dispersion in count data.

Covariates were chosen on the basis of the available literature on tsetse population dynamics and ecology [45]. In particular, thermal- and vegetation-related covariates impact on tsetse population dynamics through their direct effects on demographic parameters (birth, mortality, etc.). Moreover, because of the sampling bias and clustering of the observations in such entomological data, a spatial random effect using the Matern correlation structure was used [46]. The correlation structure was further altered to account for the temporal effects and thus resulted in a fully spatio-temporal correlation structure. Finally, model selection and, in particular, the optimal temporal lag between environmental data and tsetse apparent density was carried out by means of a likelihood-based information criterion (corrected Akaike information criterion, AICc) [43]. Each species was modelled separately and the final layer of tsetse apparent density was obtained by summing the fitted apparent densities of both *G*. *p*. *gambiensis* and *G*. *tachinoides*.

### Trypanosome infection rates in tsetse

The infection rate of tsetse flies represents the third and last layer in our risk index. A fly was considered infected if any major trypanosome species was detected (*Trypanosoma vivax*, *T*. *congolense* and *T*. *brucei*). The infection rate was modelled irrespective of tsetse species, unlike the two other models, since previous studies in the area indicated that the two species have similar infection rates [7]. It was also analyzed in the flexible framework of a generalized linear mixed model. In particular, the infection rates were investigated using a logistic regression with a random effect on the trapping site to account for spatial heterogeneity in the data. We considered temperature and host density as the main factors that influence trypanosome infection in tsetse in our study area [7]. Consequently, the model was fitted using DLST and cattle density as principal covariates and a sinusoidal function of the month when the infection status was recorded was added to the regression to account for seasonality. We also tested the same spatio-temporal correlation structure used for apparent density, which did not improve the model and was thus discarded for the sake of simplicity.

### Validation and combination of the models

For the tsetse distribution models, we used the area under the ROC curve (AUC), the specificity and the sensitivity to assess the accuracy of the fitted models. For the apparent density model, we kept one tenth of the trapping sites for each species as testing sets and we computed the percentage of variance explained by the predicted values for each models. Finally, the infection rates model was validated by computing the McFadden pseudo-R^2^ [47].

The expected apparent density of tsetse (ADT) was multiplied by the tsetse infection rates (IR) and projected into the suitable habitat (HS) to estimate the EIR (tsetse challenge). In order to have an external validation of the index and to assess its ability to predict the relationship between EIR and bovine trypanosomosis, sero-prevalence, parasitological prevalence and percentage of clinical cases were explored for various temporal (1 to 4 months) and spatial (3 to 10 km around the cattle pens) lags. It must be noted that positivity to the ELISA test corresponds either to active infections or cured, past infections. Antibodies detected with this method persist for three to five months for *T*. *vivax* [48], and about two to four months for *T*. *brucei* [49,50]. Low level of haematocrit (PCV) combined with the results of the serological status was also used as a proxy of bovine trypanosomosis: an ill animal was thus defined as a sero-positive animal with a PCV below 25%.

## Results

### Tsetse habitat suitability

NDVI-related covariates and cumulative rainfall estimates that describe health of vegetation (greenness relative density) and humidity were positively correlated with the presence of both *G*. *p*. *gambiensis* and *G*. *tachinoides*, whereas high values of temperature-related variables (DLST and NLST) lead to a low suitability index for both species. S5 Fig presents the variable contributions and response curves for the different variables. The most important variables for *G*. *p*. *gambiensis* were (in order on decreasing importance) minimum LST, minimum NDVI and mean LST whereas for *G*. *tachinoides*, altitude, mean LST and the standard deviation of NDVI were the most influential. The responses curves showed that overall, the response of both species to the different environmental variables were similar in shape. Mean LST, mean MIR and altitude were negatively correlated to suitability whereas minimum NDVI was positively correlated to suitability. However, the response of *G*. *tachinoides* to minimum NDVI was clearly less pronounced than that of *G*. *p*. *gambiensis*, confirming that the former is more xerophylous.

**Fig 2:**
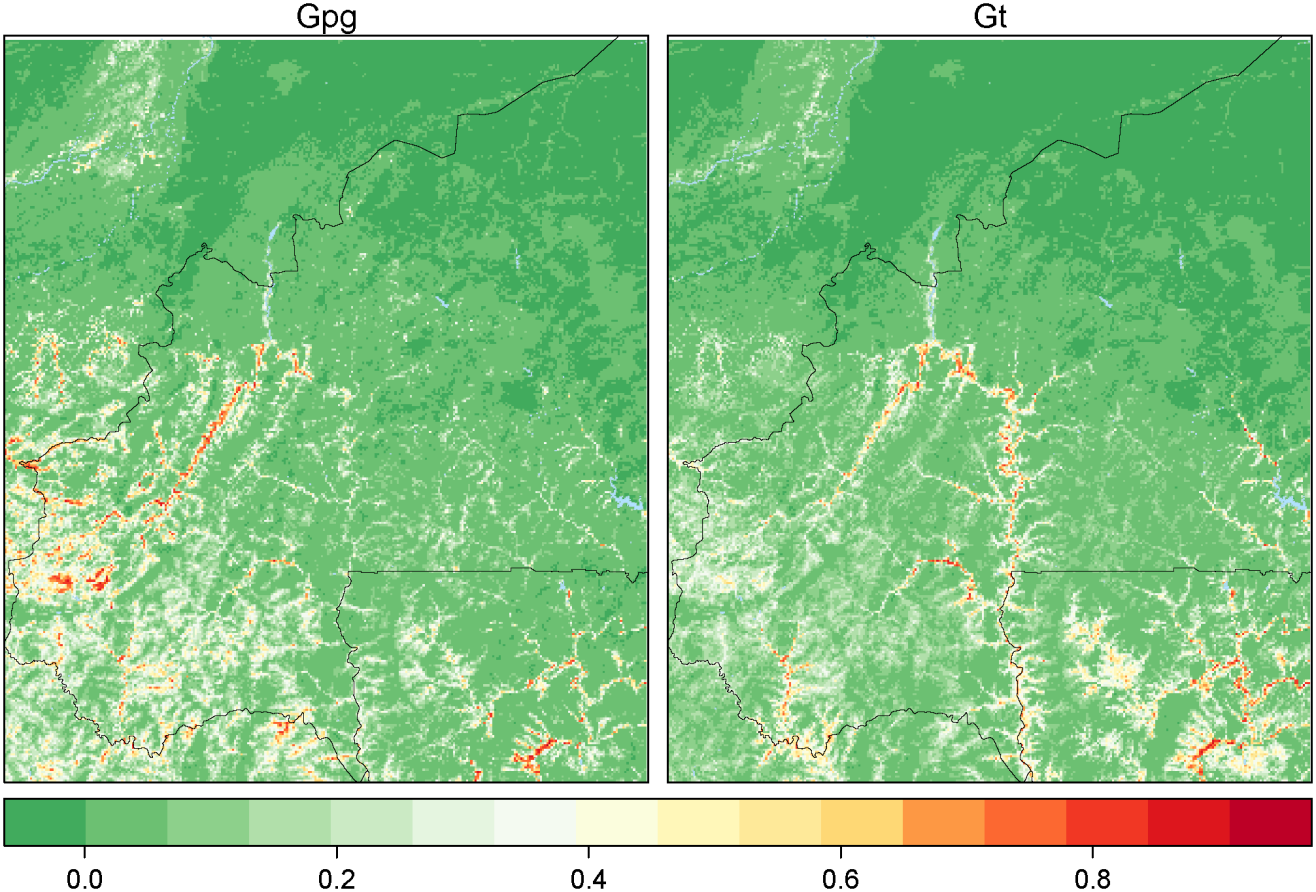
Mean predicted habitat suitability index for both species. The index varies between 0 (less suitable, green scale) and 1 (highly suitable, red scale).

**Fig 3:**
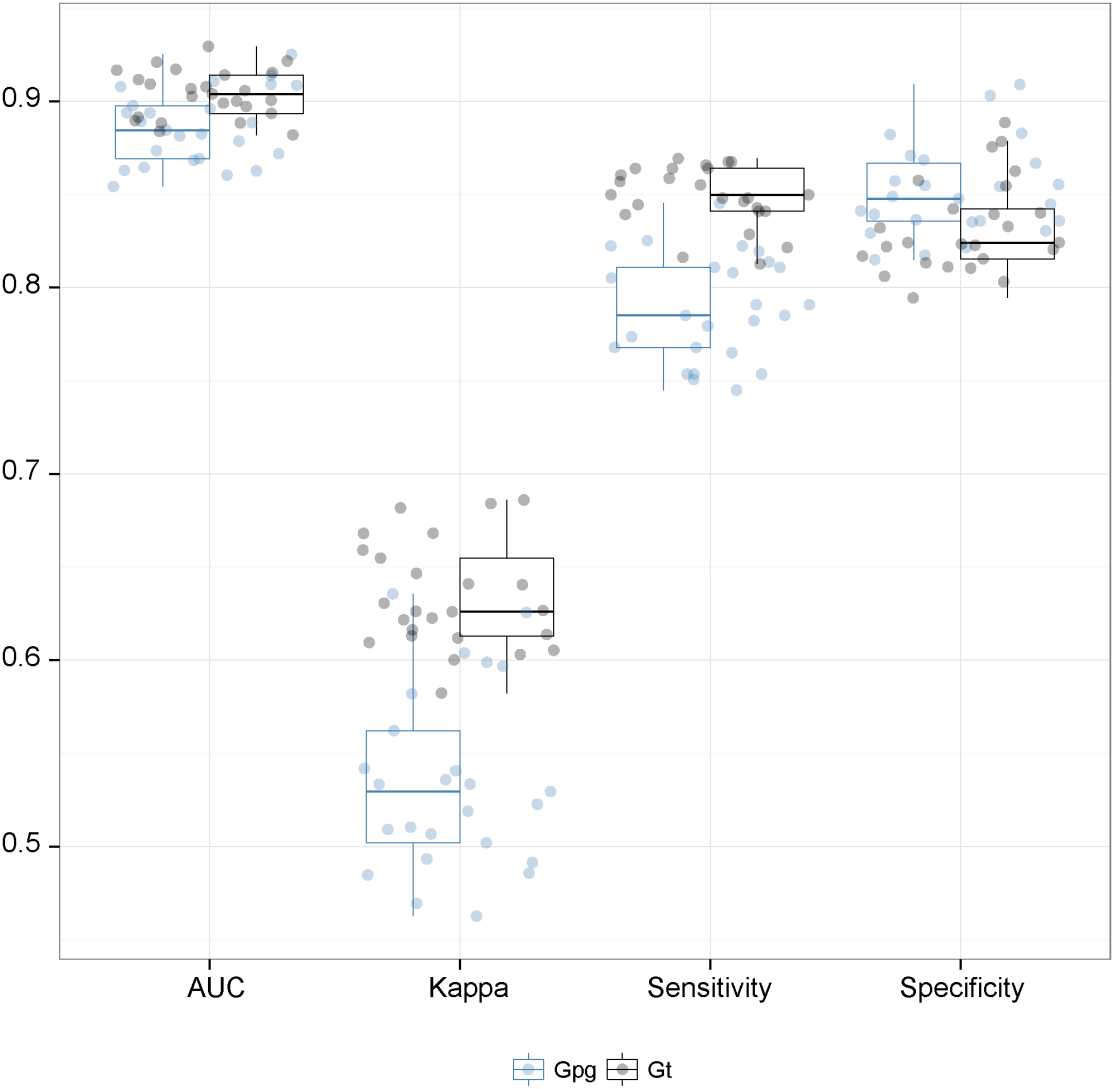
Prediction quality metrics for the habitat suitability model. AUC is the Area Under the Curve.

Fig 2 shows that hydrological network is in general highly suitable, with a wider distribution for *G*. *tachinoides* than for *G*. *p*. *gambiensis*. With the exception of a small area in western Burkina Faso, the uncertainty in the predictions was low (S6 Fig). The predictive power of each model was high with an average AUC of 0.95 (resp. 0.91) for *G*. *p*. *gambiensis* (resp. *G*. *tachinoides*) (Fig 3). The average sensitivity of the model for *G*. *tachinoides* (0.85) is higher than for *G*. *p*. *gambiensis* (0.76). The kappa statistic follows the same pattern, whereas average specificity for the habitat suitability model of *G*. *p*. *gambiensis* is higher (0.84) than that of the *G*. *tachinoides* one (0.80).

### Tsetse apparent density

The abundance of *G*. *p*. *gambiensis* was positively correlated to DLST (Table 2, p = 0.002), and the suitability index (p = 0.02) and negatively correlated to NDVI (p = 0.02). The abundance of *G*. *tachinoides* was not affected by DSLT (Table 2, p = 0.22), whereas NDVI (p<0.01) and the suitability index (p<0.01) had a positive impact. Finally, percentage of variability explained by the covariates on a test dataset was high for *G*. *p*. *gambiensis* (94%) and moderate for *G*. *tachinoides* (39%).

**Table 2:** Mixed effect negative binomial regression with spatio-temporal random effect for apparent densities of both species. ADT (Apparent Density per Trap per day), DLST (Day Land Surface Temperature), NDVI (Normalized differenced Vegetation Index), HS (Habitat suitability Index for each species). Standard error for fixed effects in brackets.

	ADT *G*. *p*. *gambiensis*	ADT *G*. *tachinoides*
Intercept	1.95 (0.99)*	0.84 (1.16)
DLST	0.06 (0.02)**	-0.02 (0.02)
NDVI	-1.88 (0.89)*	2.90 (0.95)**
HS	1.01 (0.49)*	1.47 (0.35)***

Significant codes: 0 ‘***’ 0.001 ‘**’ 0.01 ‘*’ 0.05 ‘.’ 0.1 ‘ ‘ 1

### Trypanosome infection rates in tsetse

High infection rates of tsetse were associated with high temperatures (Table 3, OR = 1.10, p < 0.01). However, a negative correlation with cattle density (domestic host) was observed in the study area (OR = 0.97, p = 0.03). The generalized linear mixed model captured the seasonality of infection rates in tsetse although with a low pseudo-R2 of 11%.

**Table 3:** Binomial random effect models for trypanosomose infection rates in tsetse (both species). DLST (Day Land Surface Temperature), Cattle_density (FAO cattle density grid), Seasonality (sinusoidal function of month when infection status was recorded). Standard error for fixed effects in brackets.

	IR
Intercept	-4.22 (0.83)***
DLST	0.10 (0.02)***
Cattle_density	-0.03 (0.01)*
Seasonality	-0.24 (0.12)*

Significant codes: 0 ‘***’ 0.001 ‘**’ 0.01 ‘*’ 0.05 ‘.’ 0.1 ‘ ‘ 1

### Entomological inoculation rates and AAT seroprevalence

**Fig 4:**
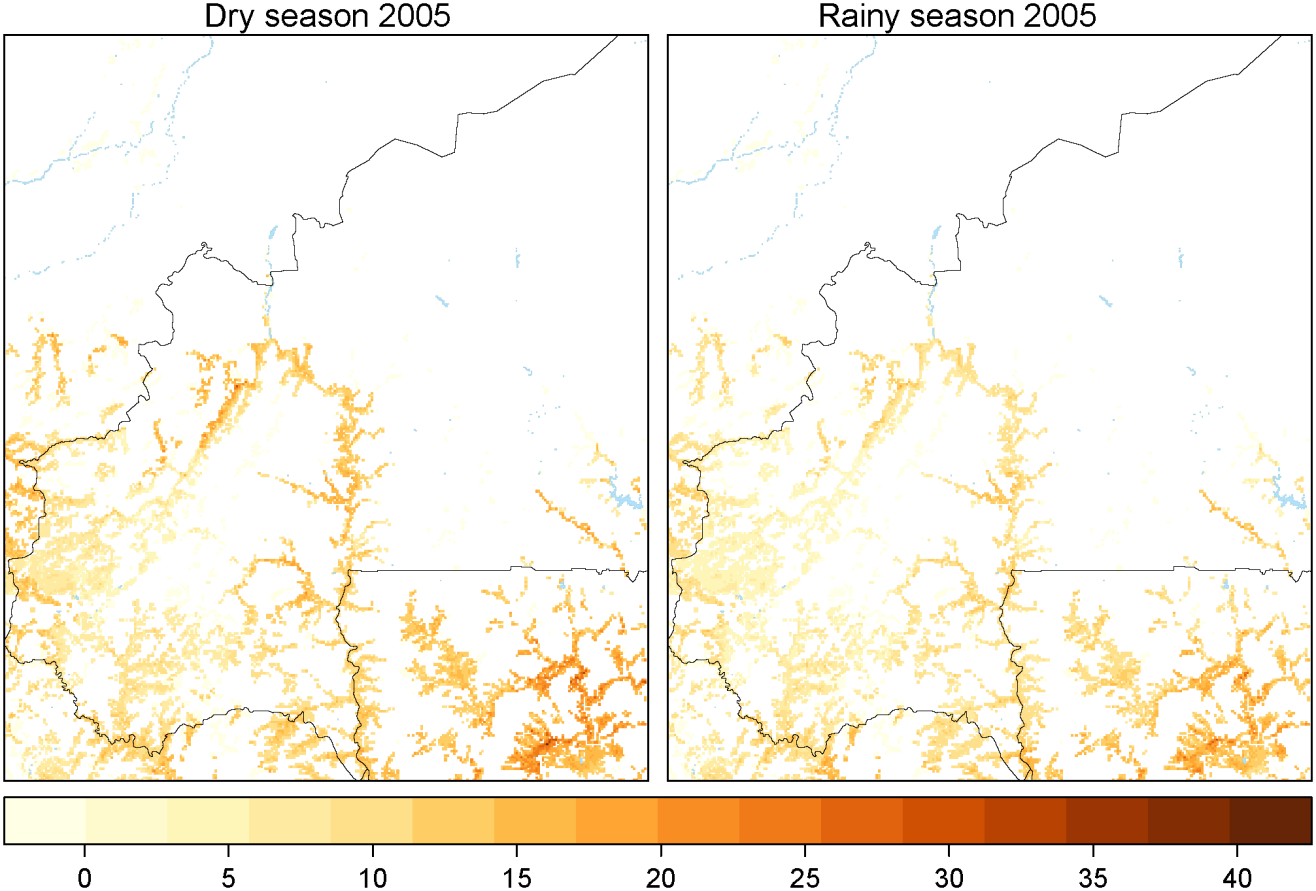
Predicted risk of bovine trypanosomosis for the dry and rainy season 2005. The risk indicator is the estimated Entomological Inoculate Rate (EIR). Darker areas in red are more at risk.

The computed EIR was high around rivers during all dry season and more widespread during the all rainy season between 2003 and 2013 (Fig 4). Optimal spatio-temporal lag for the regression of serological prevalence against EIR was obtained for a time lag of one month and a radius of 5km, which was then kept for the predictions (lowest AICc, Table 4).

**Table 4:** Optimal spatio-temporal lag for the regression model of serological and parasitological prevalences using AICc. The bold value presents the best model.

	*1 month*	*2 months*	*3 months*
Serological prevalence			
*3km*	*12656*	*12669*	*12668*
*5km*	***12631***	*12653*	*12671*
*10km*	*12648*	*12729*	*12735*
Parasitological prevalence			
*3km*	***4963***	*4966*	*4972*
*5km*	***4963***	*4965*	*4971*
*10km*	***4963***	*4965*	*4971*

**Fig 5:**
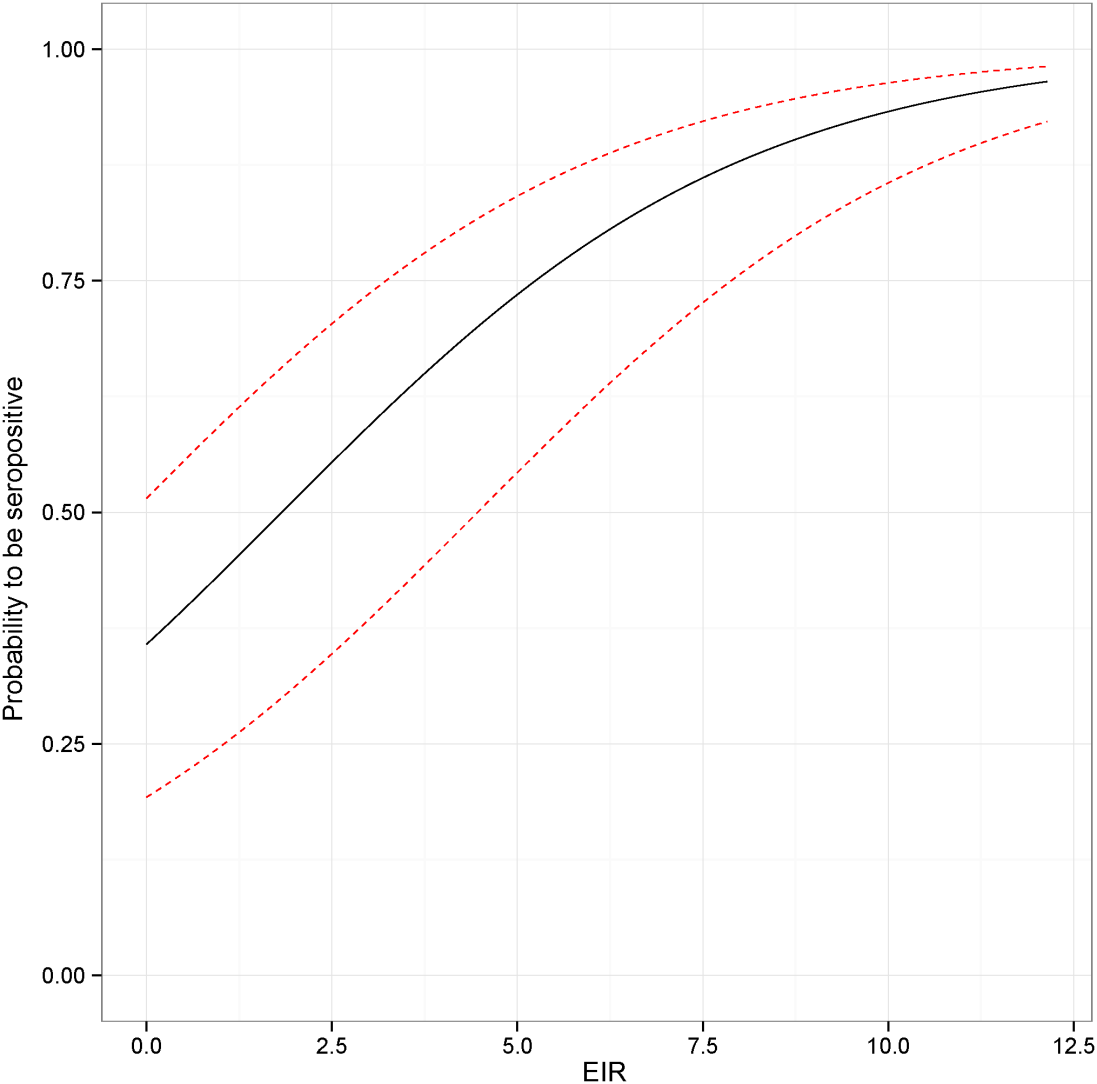
Marginal effect of the entomological inoculate rate on seropositivity probability. The confidence interval is presented as a red dashed line.

**Fig 6:**
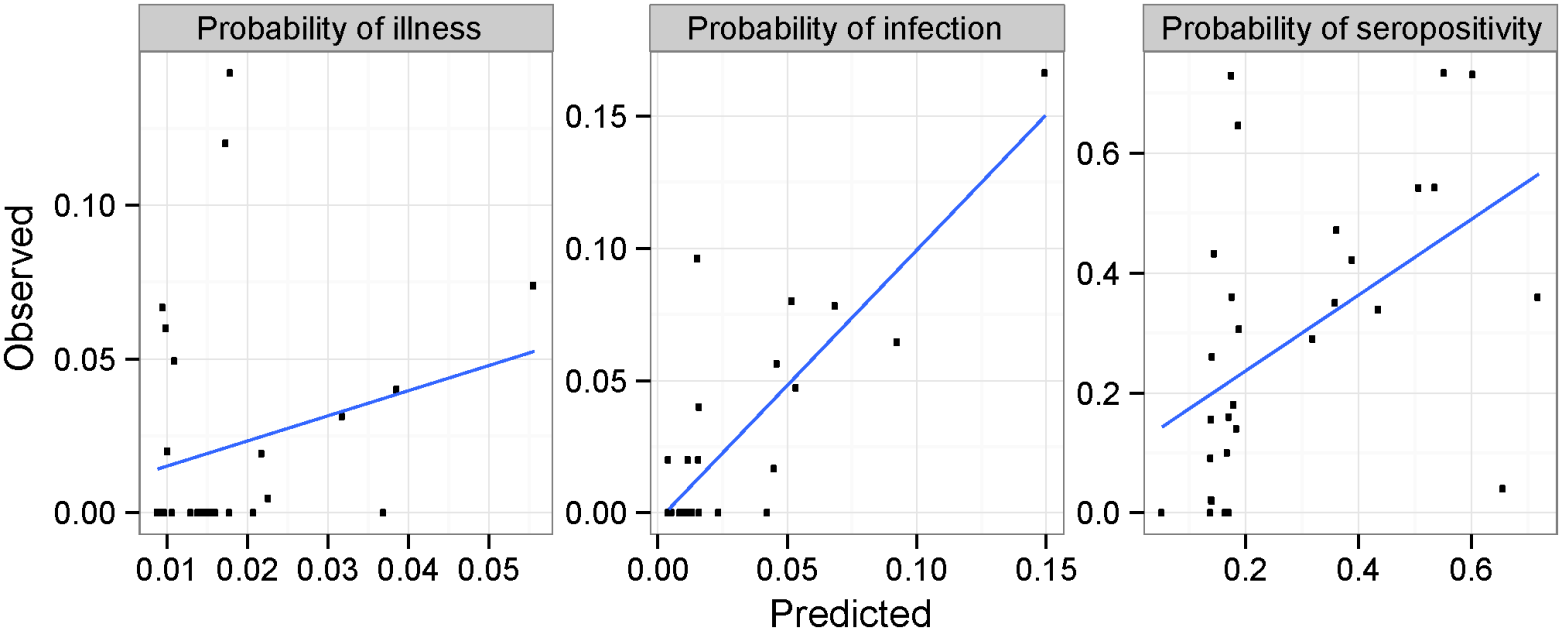
Relationship between observed and predicted disease metrics. Proportions of bovine trypanosomosis cases (illness), seropositivity, and infected animal are predicted at the village level. These predictions are made using a testing data set hold at the beginning of the analysis for prediction purpose.

For the model of seropositivity, fitted at the animal level (cattle), we used the breed (zebu/taurin/cross) of the animal and its age as co-variables. EIR had an important positive impact on sero-positivity probability (OR = 1.5, CI = 1.3–1.7) with a positive marginal effect (Fig 5 and Table 5). Observed serological prevalence aggregated at the village level showed a positive correlation with the predicted sero-prevalence (Fig 6, r2 = 22%). Optimal spatio-temporal lag for the regression of parasitological prevalence against EIR was obtained for a time lag of one month but there was no difference between the three distances tested (Table 4). For homogeneity with the serological prevalence model, we kept the 5km radius model.

**Table 5:** Logistic regression of disease metrics against EIR (entomological inoculation rate) at the cattle level. The results present the probability of an animal for being ill, having a positive parasitical status and being seropositive. The Age variable is measured in months and the Breed variable represents the breed of the animal (Taurin/Mixed/Zebu). Standard errors in brackets.

	*Illness*	*Parasitological prevalence*	*Serological prevalence*
(Intercept)	-3.99 (0.37)***	-4.91 (0.51)***	-1.33 (0.00)***
Age	0.03 (0.02)	-0.04 (0.02)**	0.01 (0.01)
EIR	0.14 (0.08).	0.19 (0.09)*	0.42 (0.07)***
Breed: cross	-0.28 (0.33)	0.90 (0.47).	-0.25 (0.16)
Breed: zebu	-0.82 (0.41)*	-0.35 (0.59)	-0.50 (0.22)**

Significant codes: 0 ‘***’ 0.001 ‘**’ 0.01 ‘*’ 0.05 ‘.’ 0.1 ‘ ‘ 1

EIR was also significantly associated to the parasitological status (Table 5, p = 0.02) and marginally to the illness status at the individual level (Table 5, p = 0.1). Older animals were also less probable to be positive to the buffy-coat test (p = 0.02). Model quality and accuracy was assessed by comparing predicted disease metrics against observed metrics aggregated at the village level on a testing dataset (25% of all data). The model had a good level of accuracy with a correlation of 67% between the observed and predicted parasitological prevalence at the village level whereas predicted illness rate at the village level was not correlated to observed values (Fig 6, r2 = 2%).

## Discussion

Overall, EIR allowed a good prediction of parasitological and serological status. Environmental parameters were shown to have an impact on both the apparent densities and infection rates of the two riverine species considered (*G*. *p*. *gambiensis* and *G*. *tachinoides*). Both species responded in a similar way to environmental parameters but with various intensities. The more important impact of minimum NDVI on *G*. *tachinoides* confirmed that this species is more xerophilous. From the spatial standpoint, the most visible and arguably predictable pattern is that AAT risk is linked to the river network, but, interestingly, a few river sections are much more risky than others, and as such they might offer priority targets for control efforts—as also previously proposed [8,9,51]. The spatio-temporal risk map of AAT presented in this study was generated and validated at a high spatial resolution and concerns a wide area. In this area, the exercise was made less challenging by the scarcity of wild fauna, leading to an endemic cycle where trypanosomes circulate mainly among livestock [52]. This cycle leads to the selection of less virulent strains that can be controlled by the combined use of curative and preventive trypanocidal drugs [53,54]. The prediction area still includes a zone where wild fauna is abundant, around the protected forest of Diéfoula, where cattle are not supposed to enter. Our model succeeded to predict a high EIR in this area and indeed, a herd that was monitored by [23] in Ouangolodougou, very close to Folonzo, during 2 years, had a very high AAT incidence (up to 20% monthly). This incidence dataset was part of the validation process. Even if farmers are not supposed to enter the protected areas, they still do so in search of better grazing areas which leads to the contact between tsetse and cattle [54]. In fact, our model probably underestimate the severity of the disease in this situation, since the strains of trypanosomes that are transmitted from wild fauna to cattle are more virulent [52].

Our analysis confirmed that higher temperatures lead to increased infection rates in tsetse. This has been attributed to increased physiological stress of tsetse associated to a higher sensitivity to infection by trypanosomes [7].

EIR was best at predicting sero-prevalence when a time lag of one month and a radius of 5km were used. The one month time lag is probably related to the time of seroconversion [55] but the best correlation with the smallest time lag tested show that the risk is quite variable in time and that our model succeeded in capturing this temporal pattern. The distance of 5km is generally considered as the ray of grazing of local sedentary herds [56], which were targeted as a priority during the various surveys. EIR is best associated to parasitological than sero-prevalence and illness status. Both parasitological and serological results can suffer from various biases. Serological diagnostic is far more sensitive than BCT and less affected by other factors like the use of trypanocide drugs, inter-recurrent diseases that may affect PCV or low parasitaemia due to trypanotolerance. On the other hand, antibodies can persist up to 13 months [57]. Thus, the probability that the animals were sampled in the area where they were actually exposed to the risk during this period is lower, even if sedentary herds were selected, due to either commercial exchanges or to past movements of the herd not necessarily considered by the farmers at the time of sampling [56]. In our study, the second category of bias is apparently more important than the former, explaining the better prediction of parasitological infection rates. Age was positively correlated to seropositivity but negatively correlated to the infection probability [58,59], which confirms that older animals develop some immunity against trypanosomes and are able to control infections better [60]. The unexpected results vis-à-vis breeds (lower seropositivity and illness in zebu than in trypanotolerant taurine cattle) might be due to confounding factors [61]. For instance, zebu are mainly present in the northern part of the study area where EIR is lower, and farmers use trypanocides more readily on zebu than on trypanotolerant cattle. Moreover, our model does not account for parasite virulence which is higher in the vicinity of protected areas [52]. Finally, trypanotolerant are generally raised under different breeding systems [62,63]. The only way this is accounted for in our model is through the selection of the grazing range in the model predicting serological and parasitological infection in cattle. However, we used the same range for all the prediction area whereas it might differ a lot between sites with different farming systems.

The present analysis is a first step in a framework for an efficient risk management approach to control climate-sensitive diseases. The methodology described in this study is generic to be applied to mitigate the risk of other vector-borne diseases through an evidence-based design of climate service mechanisms. The development and use of climate services in public health has increased recently and continues to grow, especially in the context of a changing climate [50]. More specifically, an optimal transfer of bovine trypanosomosis risk and incentives for disease control by livestock owners can be achieved through the design of index-based animal disease insurance [64].

This analysis has also consequences for Human African Trypanosomosis (HAT) commonly known as sleeping sickness. Indeed, it has been suggested that climate change is likely to impact the risk of HAT in Africa [65]. However, despite the advocacy for a One Health approach [66,67] to control such diseases, climate services to mitigate both the risk of HAT and AAT have not been designed yet. The model developed in this study can be scaled up across Africa [68] and might thus serve as the basis for a spatio-temporal model of sleeping sickness risk in Africa.

The approach developed in this study enjoys a degree of flexibility because modelling separately each component of the risk (EIR), state-of-the-art methodology for each compartment can be used. However, there are some caveats when using this "one layer—one model" approach; which are related to the increasing uncertainty at each step of the modelling process [37]. This uncertainty impacts on the estimated EIR directly. Therefore, further work is needed to develop a more robust approach to design spatio-temporal risk maps based on sparse entomological data and to evaluate these maps so that they can potentially serve as early warning systems [69].

Another important aspect to keep in mind regarding AAT risk is the role of mechanical transmission [70]. In fact, it has been suggested that, when tsetse population become sparser or disappear, other biting flies like Tabanides or Stomoxines could maintain AAT transmission, also through episodic epidemics similar to those observed in South America for *T*. *vivax*. This can constitute a potential bias in our model that accounts only for cyclical transmission. Indeed, Tabanides, which are very common in the study area, have been shown to transmit *T*. *vivax* at incidence rates as high as 63% (*Atylotus agrestis*) and 75% (*Atylotus fuscipes*) within 20 days, and *T*. *congolense* at a cumulative incidence rate of 25% (*A*. *agrestis*) in experimental conditions [71].

## Supporting Information

S1 Fig Geographical location of entomological data. Presence (black dot) and absence (red dot) data for *G*. *palpalis gambiensis* in the study area.
(TIF)

S2 Fig Geographical location of entomological data. Presence (black dot) and absence (red dot) data for *G*. *tachinoides* in the study area.
(TIF)

S3 Fig Geographical location of sampled cattle. The serological prevalence of cattle herds is presented with proportional sizes of circles.
(TIFF)

S4 Fig Bias grids for the MaxEnt model. In each panel, a bias grid with corresponding smoothing parameter (dispersal in km).
(TIF)

S5 Fig Variables contribution and responses curves of the main variables in the MaxEnt models. (TIF)

S6 Fig Uncertainty grid for the habitat suitability index model. Areas in red are the most inaccurate and should be interpreted with care.
(TIF)

S1 File Tsetse apparent density in a csv format. For the species column, gpg = G. palpalis gambiensis and gt = G. tachinoides.
(CSV)

S2 File Tsetse infection rates in a csv format. (CSV)

S3 File Data on bovine trypanosomosis. Serological, parasitological and illness statuses are provided at the animal level.
(CSV)
