# Supplementary figures and images for: A Spatio-temporal Model of African Animal Trypanosomosis Risk
Source: PLoS Negl Trop Dis. 2015 Jul 8;9(7):e0003921. doi: 10.1371/journal.pntd.0003921 (PMC4495931; doi:10.1371/journal.pntd.0003921)

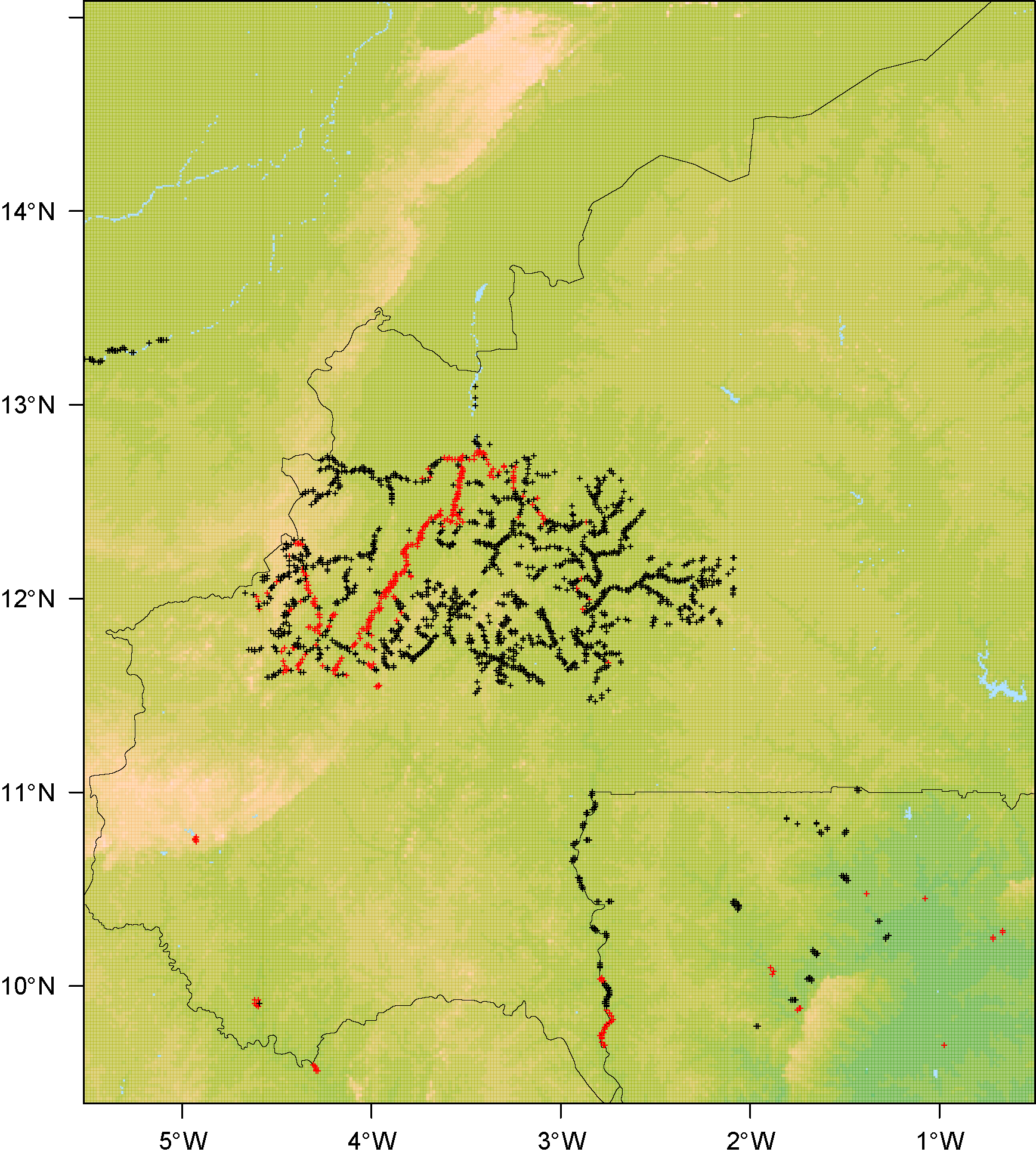

Supplement: S1 Fig — Presence (black dot) and absence (red dot) data for G. palpalis gambiensis in the study area. (TIF) [file pntd.0003921.s001.tif]

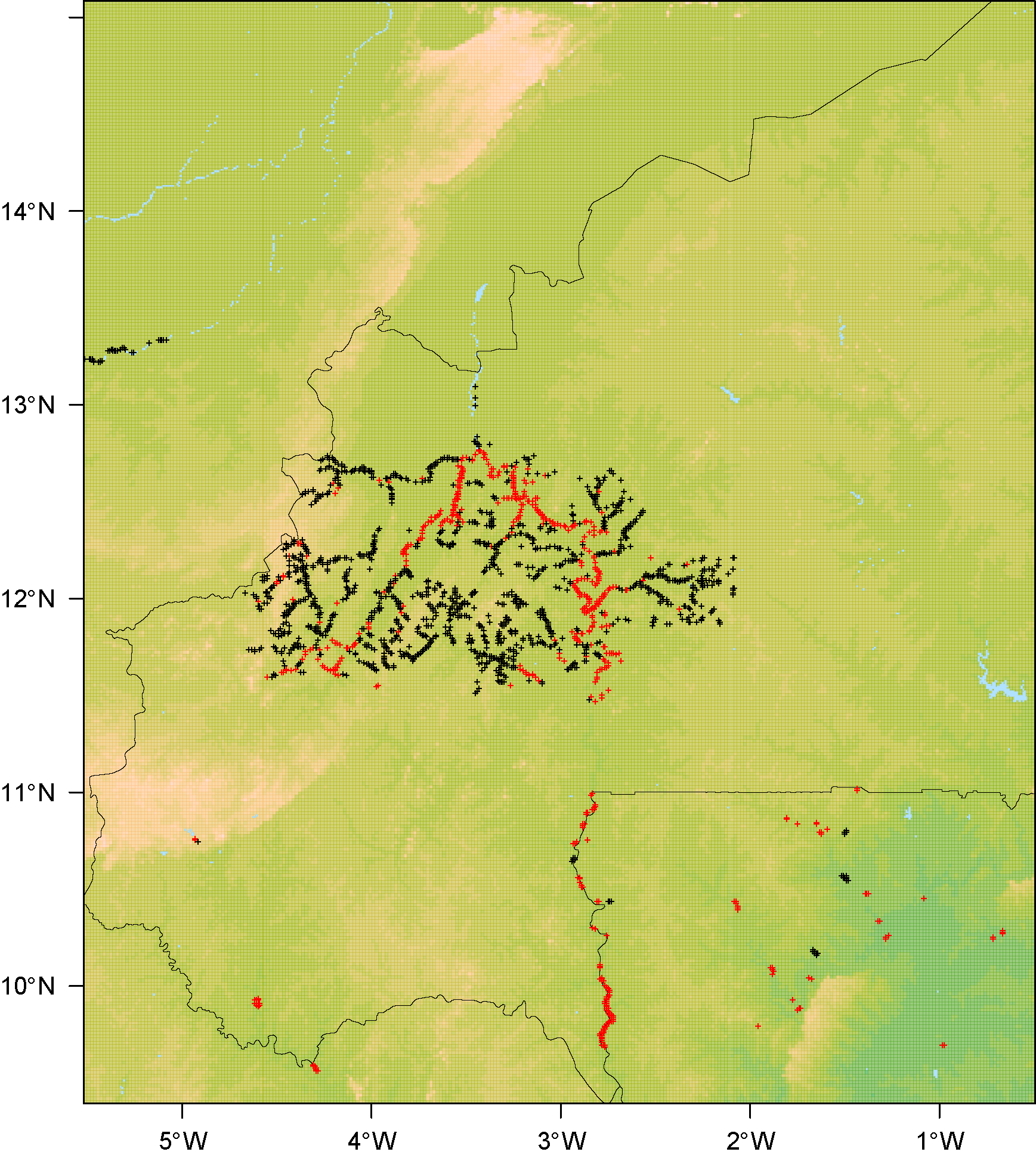

Supplement: S2 Fig — Presence (black dot) and absence (red dot) data for G. tachinoides in the study area. (TIF) [file pntd.0003921.s002.tif]

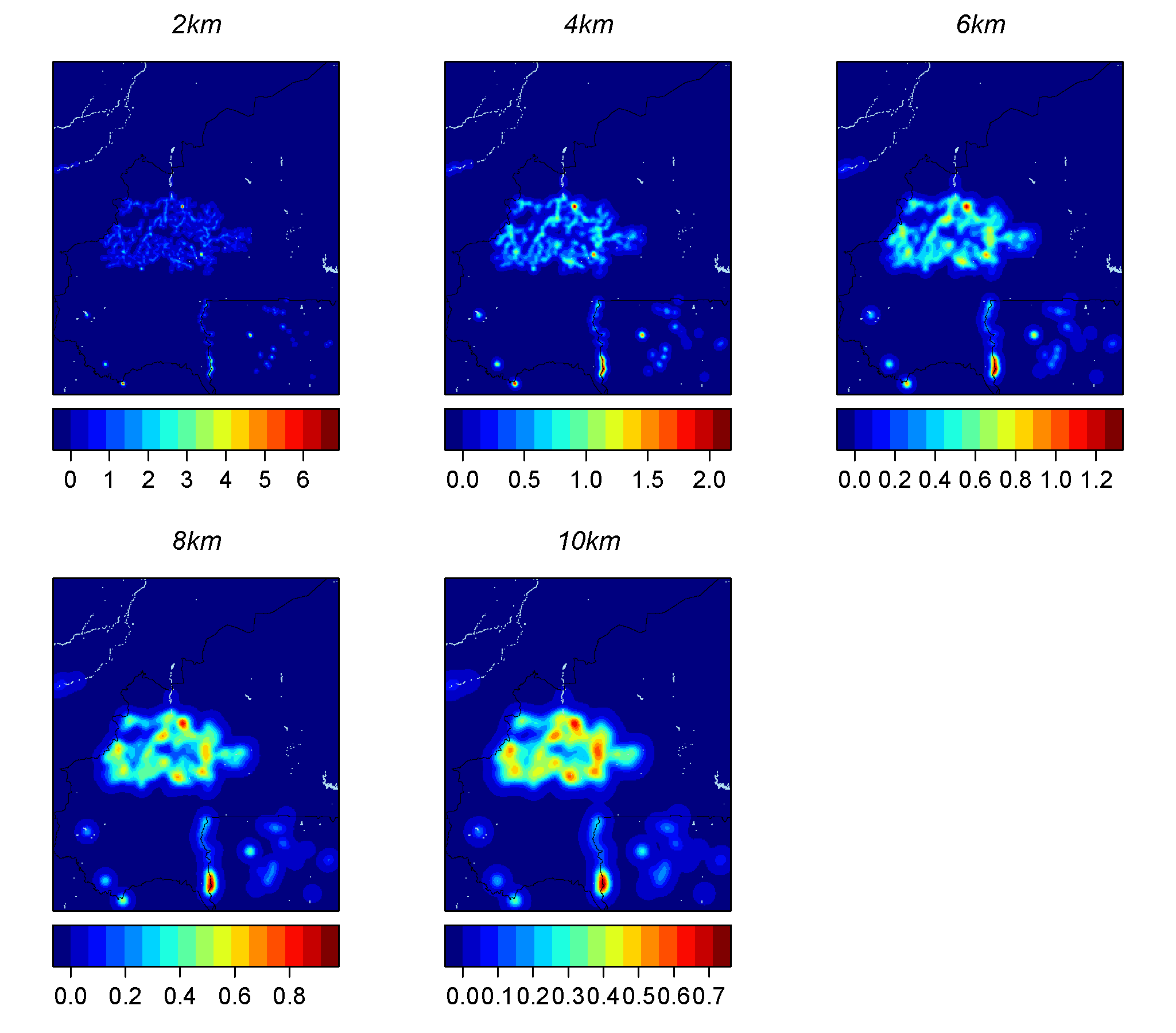

Supplement: S4 Fig — In each panel, a bias grid with corresponding smoothing parameter (dispersal in km). (TIF) [file pntd.0003921.s004.tif]

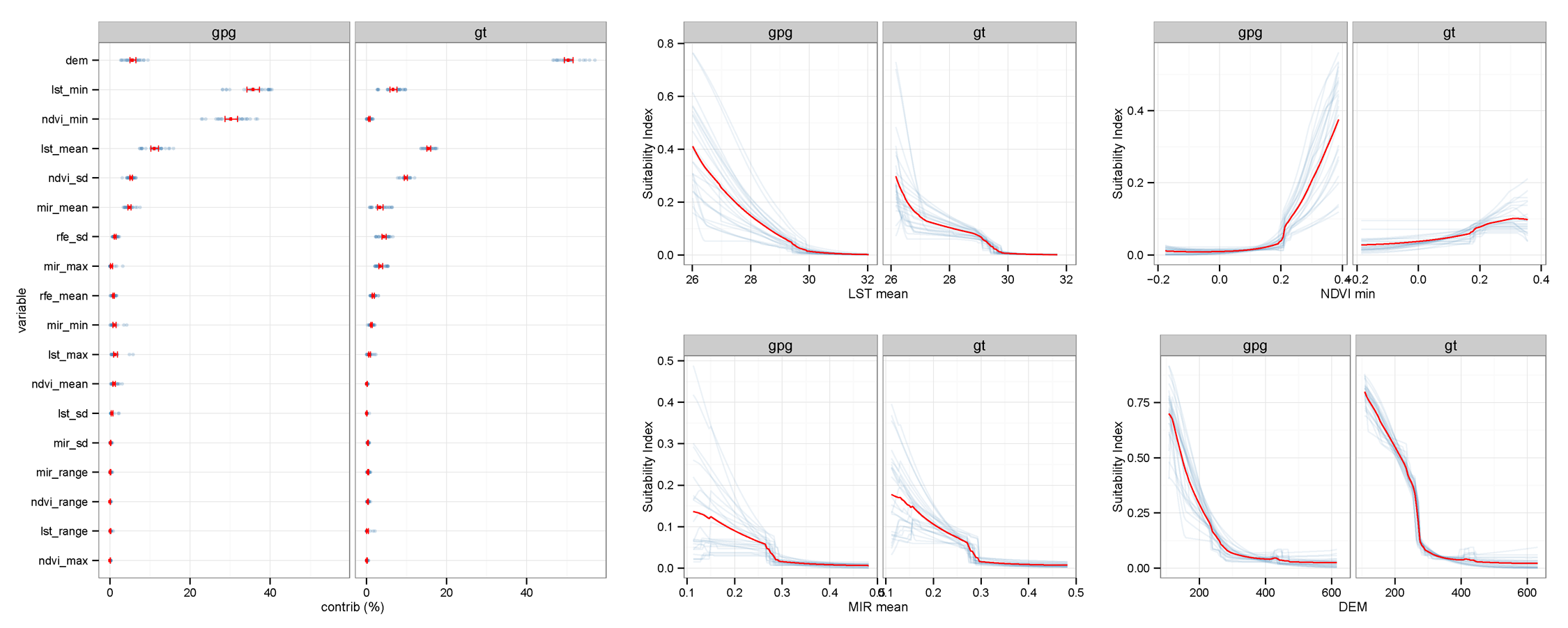

Supplement: S5 Fig — (TIF) [file pntd.0003921.s005.tif]

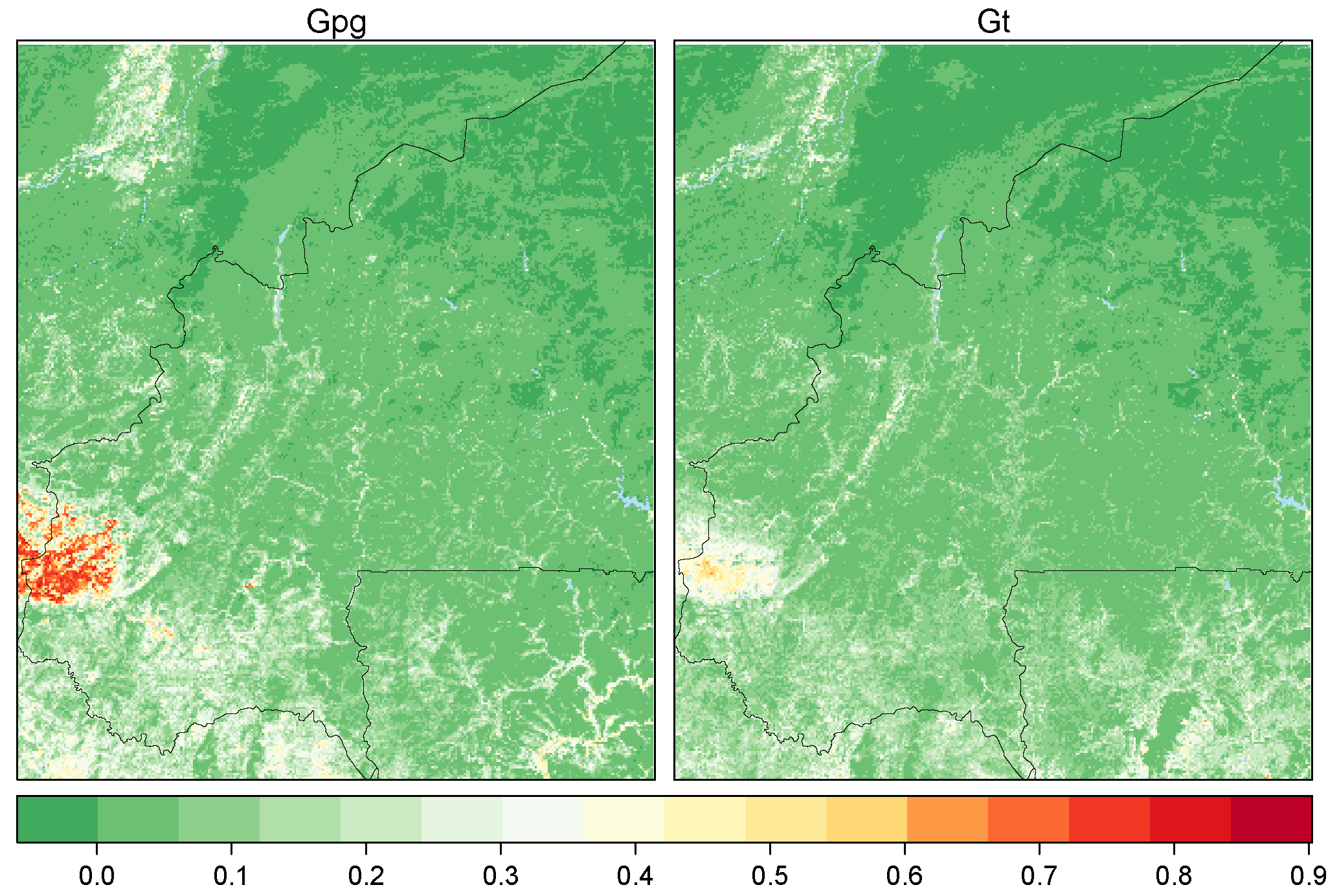

Supplement: S6 Fig — Areas in red are the most inaccurate and should be interpreted with care. (TIF) [file pntd.0003921.s006.tif]
